# On a generalizable approach for sample size determination in Bayesian *t* tests

**DOI:** 10.3758/s13428-025-02654-x

**Published:** 2025-03-31

**Authors:** Tsz Keung Wong, Jorge N. Tendeiro

**Affiliations:** 1 Department of Methodology & Statistics, Tilburg University, Warandelaan 2, 5037 AB Tilburg, The Netherlands; 2 Graduate School of Advanced Science and Engineering, Hiroshima University, Hiroshima, Japan

**Keywords:** Bayes factor, Design analysis, Sample size determination, Power analysis

## Abstract

The Bayes factor is often proposed as a superior replacement to *p* values in testing null hypotheses for various reasons, with the availability of many user-friendly and easily accessible statistical software tools facilitating the use of Bayesian tests. Meanwhile, Bayes factor design analysis (BFDA), the counterpart of power analysis, is also proposed to ensure the maximum efficiency and informativeness of a study. Despite tools for conducting BFDA being limited and mostly relying on Monte Carlo methodology, methods based on root-finding algorithms have been recently developed (e.g., Pawel and Held, 2025), overcoming weaknesses of simulation approaches. This paper builds on these advancements by presenting a method generalizing the existing approach for conducting BFDA for sample size determination in *t* tests. The major advantage of the current method is that it does not assume normality of the effect size estimate, allowing more flexibility in the specification of the design and analysis priors. We developed and showcase a user-friendly Shiny app for facilitating the use of BFDA, illustrated with an empirical example. Furthermore, using our method, we explore the operating characteristics of the Bayes factors using various priors.

## Introduction

In Neyman–Pearson-based null hypothesis significance testing (NHST), a point null hypothesis is tested against an alternative hypothesis with a specific hypothesized value (Neyman & Pearson, 1933). A priori power analyses are needed to control the error rates of the test and determine a minimum required sample size (Cohen, 1992). Despite the ubiquitous use of NHST in the field of psychology, null hypothesis Bayesian testing (NHBT) has been advocated and proposed as a better replacement (e.g., Wagenmakers, 2007). Notably, the development of the Bayesian *t* test by Rouder et al. (2009), along with the *BayesFactor* R package and statistical software JASP (JASP Team, 2024), has had an influential impact in the field. The accessibility of these user-friendly statistical tools allows applied researchers to conduct Bayesian *t* tests easily.

Meanwhile, the counterpart of power analysis in Bayesian statistics is still under development. The limited options currently available contrast with the plethora of readily and user-friendly available tools for conducting power analysis in NHST, such as G*Power (Faul et al., 2007) and the R packages *pwrss* (Bulus, 2023) and *pwr* (Champely, 2020). The underlying reason for the lack of tools to conduct Bayesian power analyses might be that the concept of controlling long-term error rates is inconsistent with the Bayesian school of thought. The aim of Bayesian testing is to quantify and communicate the relative strength of evidence via the Bayes factor (BF; Kass and Raftery 1995) rather than to make a behavioral dichotomous decision regarding the cost of incorrect testing decisions (type I and type II errors) in the long run. Furthermore, the mathematical complexity involved in deriving the distribution of the BF might hinder the development of closed-form methods for sample size determination. Nonetheless, in the situation of testing for the mean of a normal model with known variance, Pawel and Held (2025) derived closed-form solutions for sample size determination in Bayesian z-tests. These authors offer the *bfpwr* R package to implement the analysis in practice, building on the work of De Santis (2004) and Weiss (1997).

The mathematical challenge required to move from a Bayesian power analysis based on the z-test to a Bayesian power analysis based on the *t* test leads researchers to resort to Monte Carlo methodology to perform the required computations. The Monte Carlo simulation approach generally requires intensive computational power and some basic programming skills from the users. For instance, the sample size determination for Bayesian independent samples *t* tests, Welch’s tests, and ANOVA with informative hypothesis and fractional BF is developed within the *SSDbain* package (Fu et al., 2020, 2022). Additionally, Schönbrodt and Wagenmakers (2018) explored the Bayes factor design analysis (BFDA) framework, which evaluates the efficiency and informativeness of a study using a fixed-*n* design or a sequential design, where data is collected until a certain threshold of BF is reached. Unlike frequentist power analysis, BFDA focuses on designing experiments that yield informative results while minimizing resource use. Thus, the objective of BFDA is to determine the smallest possible sample size that ensures a high probability of compelling evidence and a low probability of misleading evidence. For practical applications, a tutorial on BFDA, along with a Shiny app and the *BFDA* R package, is available in Stefan et al. (2019). Recently, Pawel and Held (2025) utilized a root-finding algorithm to conduct BFDA in *t* tests using analytical solutions without requiring simulations. This root-finding approach is superior to the existing Monte Carlo method, as it greatly reduces computational time and eliminates the variability of results due to Monte Carlo error. Consequently, this method avoids the mathematical difficulties and computational demands associated with simulations.

Building on the foundation of the BFDA framework and the root finding algorithm, this article aims to develop a generalizable method for sample size determination in Bayesian *t* tests, *without the assumption of normality on effect size estimates* (which is the essential assumption in the method by Pawel and Held, 2025). Our paper offers additional insights compared to existing methods in several ways. First, conducting BFDA is more flexible with our approach. In essence, instead of utilizing analytic solutions relying on the assumption of normality, we use numeric integration based on prior predictive distributions (we explain how in this paper). This approach allows us to calculate the probabilities of compelling and misleading evidence using any proper prior, such as the scaled *t*-distribution, normal distribution, or a non-local prior (Pramanik & Johnson, 2024). Moreover, our method has the potential to be extended to all BFs obtained by numeric integration regardless of the normality assumption.

Second, a user-friendly Shiny app has been developed to facilitate BFDA for determining sample sizes and evaluating the informativeness of studies with fixed sample sizes and (un)balanced designs. In cases of limited resources or the use of existing datasets, having a sufficiently large sample size to ensure the informativeness of the study might not be feasible, and unbalanced designs become unavoidable. To address this, we extend the existing method to allow the evaluation of a study’s informativeness for a fixed sample size with an unbalanced design.

Third, we examine how different prior specification influences the operating characteristics of BFs in independent samples Bayesian *t* tests. The operating characteristics of the BF include properties such as the probability of compelling and misleading evidence, which are analogous to the power and false-positive/negative rates in NHST, respectively. From a frequentist’s perspective, we found that independent samples Bayesian *t* tests perform better than NHST in controlling false-positive rates with the conventional levels. Our results are aligned with existing findings (Kelter, 2021; Pawel & Held, 2025). More specifically, compelling BFs for the alternative hypothesis relative to the null hypothesis are almost always associated with significant *p* values with the conventional significance level α = .05. However, significant *p* values do not necessarily correspond to compelling BFs.

The target audience of this paper is the applied researchers who wish to conduct Bayesian *t* tests in their research but are unaware of BFDA as a replacement for power analysis. Additionally, the present paper targets researchers already familiar with Bayesian testing and BFDA and wish to conduct BFDA in their studies. Introductory material on the BF can be found in Kruschke and Liddell (2017), Tendeiro et al. (2024), and Wagenmakers et al. (2017). While this article summarizes BFDA in a later section, detailed information on BFDA is available in Schönbrodt and Wagenmakers (2018) and Stefan et al. (2019). The article is organized as follows. First, we provide an introduction to the BF in the context of *t* tests within the framework of BFDA. In particular, we elaborate on the prior distributions considered under the alternative hypothesis, and the difference between the analysis prior and the design prior. Subsequently, the concept of prior predictive distributions is introduced, which is crucial for our method for sample size determination. Second, we elaborate on the technical details of our method, and we compare our method with that by Pawel and Held (2025). Third, the procedure for conducting BFDA is introduced and our Shiny app is showcased with an empirical illustration. Fourth, we investigate the operating characteristics of Bayesian independent samples *t* tests with a focus on false-positive rates and power. Lastly, we discuss our main results and future research avenues.

## Null hypothesis Bayesian testing and Bayes factor design analysis

### The Bayes factor

The BF, which is the Bayesian test statistic, was originally developed by Harold Jeffreys (1939). It can be shown that (e.g., Etz & Vandekerckhove, 2017) :

1 $$\underset{\text{Posterior odds}}{\underbrace{\frac{p\left({H}_{1}|Data\right)}{p\left({H}_{0}|Data\right)}}}=\underset{{BF}_{10}}{\underbrace{\frac{p\left(Data|{H}_{1}\right)}{p\left(Data|{H}_{0}\right)}}} \times \underset{\text{Prior odds}}{\underbrace{\frac{p\left({H}_{1}\right)}{p\left({H}_{0}\right)}}}$$

The BF is the first term on the right-hand side of Eq. (1), which is the ratio of the likelihood of the data under the alternative hypothesis ${H}_{1}$ over the one under the null hypothesis ${H}_{0}$. We use the subscript notation BF_10_ to indicate the order of the relative comparison between both hypotheses. BF_10_ quantifies the evidence for the alternative hypothesis relative to the null hypothesis, whereas BF_01_ indicates the evidence for the null hypothesis relative to the alternative hypothesis, with BF_01_ = 1/ BF_10._ A BF_10_ of 5 suggests that the data are five times more likely to be observed under the alternative hypothesis than the null hypothesis. Alternatively, from Eq. (1), we can see that BF_10_ equals the ratio of the posterior odds over the prior odds, indicating how the relative degree of belief in either hypothesis should be updated in light of the observed data. Given a BF_10_ of 5, a rational agent should update their relative belief in favor of the alternative hypothesis by a factor of 5-to-1, in light of the observed data.

Qualitative interpretation of the magnitude of a BF has been proposed by various researchers (e.g., Jeffreys, 1961; Kass & Raftery, 1995; Lee & Wagenmakers, 2014). However, such labels (such as “substantial”, “strong”, or “decisive”) are somewhat arbitrary, potentially leading testers to interpret BFs mechanically. Therefore, it is recommended that testers define a threshold for what constitutes compelling evidence before data collection, tailored to each unique research scenario and testing problem, and report the exact value of the BF. For illustrative purposes, in this paper, the bound for compelling evidence is defined as ${BF}_{b}=10$. The subscript “b” indicates the bound for BF_10_ and BF_01_ simultaneously. Thus, when BF_10_ is bigger than 10, there is compelling evidence for the alternative relative to the null. Similarly, if BF_01_ is bigger than 10 (or BF_10_ < $\frac{1}{10}$), there is compelling evidence for the null relative to the alternative. If BF_10_ is between $\frac{1}{10}$ and 10, the evidence is inconclusive either way.

### Probabilities of compelling and misleading evidence

The purpose of BFDA is to optimize the design of an experiment in order to make strong inferences (Schönbrodt & Wagenmakers, 2018). The idea is to ensure a sufficiently high probability of obtaining compelling evidence, while simultaneously having a sufficiently low probability of obtaining misleading evidence. This approach optimizes experiments to produce informative results while minimizing the required sample size. Table 1 outlines key concepts to consider when conducting BFDA. Compelling evidence refers to situations where the data strongly support the true hypothesis at hand. Conversely, misleading evidence occurs when the data strongly support the incorrect hypothesis (Royall, 2000; Schönbrodt & Wagenmakers, 2018). Thus, the purpose of BFDA is to determine the minimal sample size to ensure that the probabilities of obtaining true-positive evidence and true-negative evidence – denoted as $p({BF}_{10}>10|{H}_{1}$) and $p({BF}_{01}>10|{H}_{0}$) given ${BF}_{b}=10$, respectively – are sufficiently high, while the probabilities of false-positive and false-negative evidence – $p({BF}_{10}>10|{H}_{0}$) and $p({BF}_{01}>10|{H}_{1}$), respectively – are sufficiently low.

**Table 1:** The probabilities of compelling and misleading evidence

	Evidence for H0 relative to H1	Evidence for H1 relative to H0
H0true	True-negative evidence (TNE): $p\left({BF}_{01}>b|{H}_{0}\right)$	False-positive evidence (FPE):$p\left({BF}_{10}>b|{H}_{0}\right)$
H1true	False-negative evidence (FNE):$p\left({BF}_{01}>b|{H}_{1}\right)$	True-positive evidence (TPE):$p\left({BF}_{10}>b|{H}_{1}\right)$

The concepts above within the framework of BFDA might be probabilistically similar to the type I (α) and type II (β) error rates (i.e., their complements, true-negative rate and power) in Neyman–Pearson-based NHST. However, it is important to note that the latter involves making a dichotomous decision and statistical inference with regard to the error rates in the long run. In contrast, the former aims to help researchers design informative and efficient experiments without making a behavioral decision. These probabilities of misleading and compelling evidence only represent the operating characteristics of the study using the BF and are not relevant in making inferences once the data is collected (Schönbrodt & Wagenmakers, 2018).

### Bayesian *t* tests and the analysis prior

In the context of the one-sample *t* test, the null hypothesis is usually a point hypothesis such as

$${H}_{0}: \delta =0.$$

The parameter $\delta$ is the effect size defined as $\delta = \frac{\mu}{\sigma}$, where $\mu$ and $\sigma$ are the population mean and the standard deviation, respectively. The likelihood of the data (or, equivalently, of a specific *t*-value) under the null hypothesis, $p\left(t|{H}_{0}\right)$ is given by ${T}_{v}\left(t|0\right)$ , where ${T}_{v}$ is the *t* likelihood function with $v$ degrees of freedom and non-centrality parameter equal to 0 (thus, in effect, ${T}_{v}$ is a central *t* distribution). As for the alternative hypothesis, a two-sided hypothesis is used as an illustrative example:

$${H}_{1}: \delta \ne 0.$$

The likelihood of the data under the alternative is (Gronau et al., 2020):

2 $$p\left(t|{H}_{1}\right)=\int {T}_{v}\left(t|\sqrt{N}\delta \right){\pi }_{a}\left(\delta \right)d\delta .$$

This likelihood is the marginalized likelihood of a specific *t*-value across all possible values of $\delta$, with the prior density function of $\delta$ being denoted as ${\pi }_{a}(\delta )$. The subscript “a” indicates the prior density function being used for the calculation of the BF in the statistical analysis once the data is collected. Within the framework of BFDA, this density is called the *analysis prior*. Gronau et al. (2020) showed that by placing the so-called Jeffreys prior (Jeffreys, 1961) on σ under both hypotheses, the BF becomes:

3 $${BF}_{10}\left(t\right)= \frac{\int {T}_{v}\left(t|\sqrt{N}\delta \right){\pi}_{a}\left(\delta \right)d\delta }{{T}_{v}\left(t|0\right)}.$$

Researchers can compute a BF by means of numeric integration by inserting the prior density of interest for ${\pi }_{a}\left(\delta \right)$ (Gronau et al., 2020). Equation (3) can be readily extended to independent samples *t* tests with $v= {n}_{1}+{n}_{2}-2$ based on the standardized mean difference $\delta = \frac{{\mu }_{1}-{\mu }_{2}}{\sigma }$ with the assumption of equal variances. The formula to compute the BF becomes

4 $${BF}_{10}\left(t\right)= \frac{\int {T}_{v}\left(t|\sqrt{\frac{{n}_{1}{n}_{2}}{{n}_{1}+{n}_{2}}}\delta \right){\pi}_{a}\left(\delta \right)d\delta }{{T}_{v}\left(t|0\right)}.$$

### Prior distributions considered

Bayesian testing requires specifying a prior distribution for all possible values of $\delta$. The selection of a prior distribution formalizes a substantive research hypothesis into a statistical model that reflects the researcher’s expectations regarding the effect size (Vanpaemel & Lee, 2012). Moreover, this prior distribution reflects the researcher’s epistemic uncertainty and lack of precise knowledge about the effect size (Pek & Park, 2019). Using a prior distribution also accounts for the effect size heterogeneity, considering heterogeneity is prevalent in meta-analysis (Linden & Hönekopp, 2021; Van Erp et al., 2017).

Following Rouder et al. (2009) and Gronau et al. (2020), the Cauchy distribution and its generalized form, a scaled *t*-distribution, are considered:

5 $$\pi \left(\delta \right)= \frac{1}{\lambda}{T}_{k}\left(\frac{\delta -{\mu}_{\delta}}{\lambda}\right).$$

An informed prior can be specified by selecting the location parameter ${\mu }_{\delta }$ , scaling parameter $\lambda$, and the degrees of freedom $k$. When ${\mu}_{\delta}=0$ and $\lambda =1$ with $k=1$, the scaled *t*-distribution becomes the Cauchy distribution. The normal distribution is also considered, with the mean and the variance of the effect size being denoted as ${\mu}_{\delta}$ and ${\sigma}_{\delta}^{2}$, respectively. The scaled *t*-distribution approaches the normal distribution as the number of degrees of freedom approaches infinity. Lastly, we also consider the normal moment prior proposed by Johnson et al. (2023), which is a *non-local* prior (Johnson & Rossell, 2010). Non-local priors are argued to more accurately reflect prior beliefs compared to local priors such as the Cauchy distribution (Pramanik & Johnson, 2024). The density function is defined as:

6 $$\pi \left(\delta \right)=\frac{{\left(\delta -{\mu}_{\delta}\right)}^{2}}{\sqrt{2\pi }{\tau }^{3}}{e}^{-\frac{{\left(\delta -{\mu}_{\delta}\right)}^{2}}{2{\tau }^{2}}}.$$

**Fig. 1:**
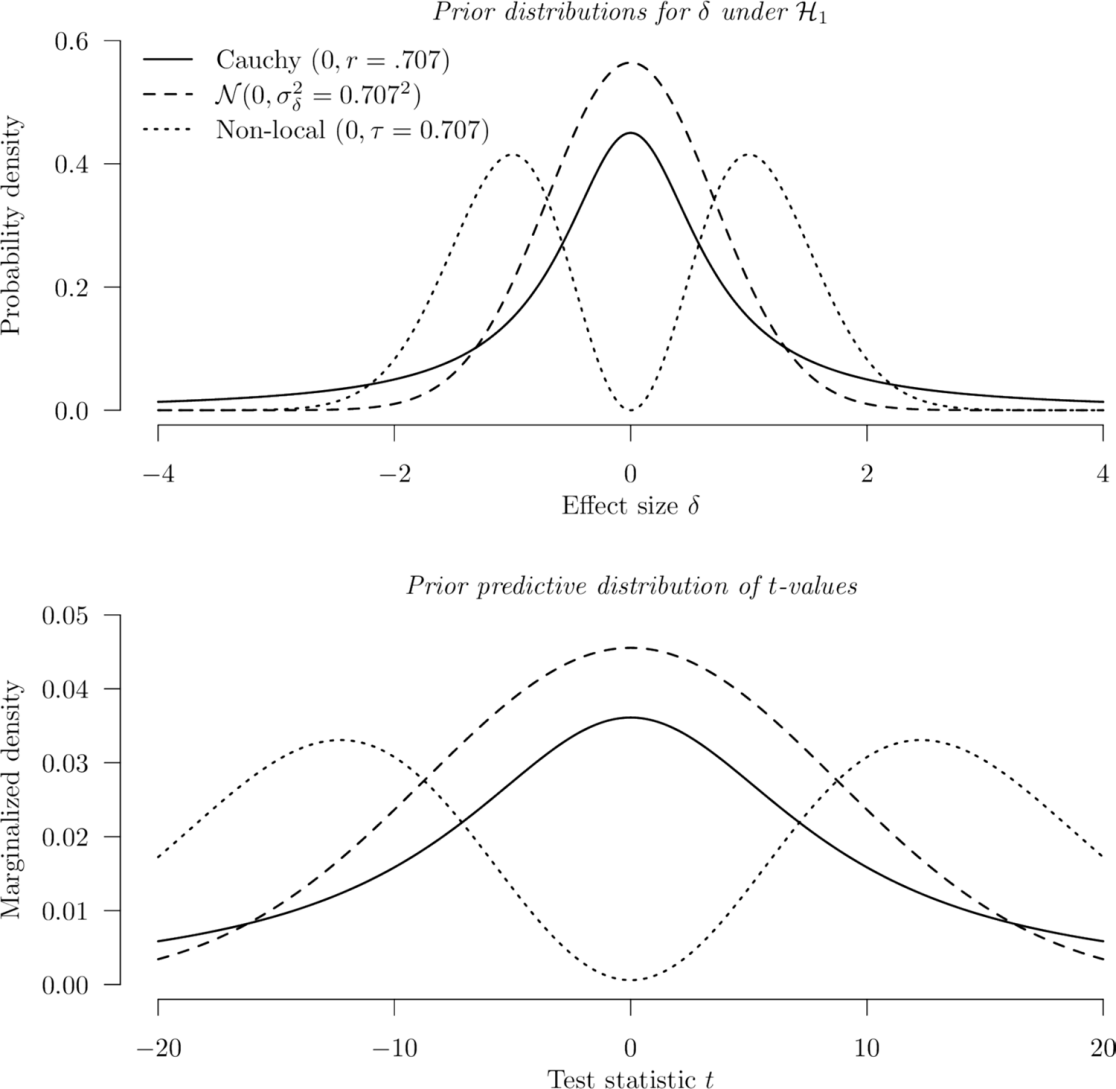
The prior distributions on $\updelta$ (***top panel***) and the associated prior predictive distributions on *t*-values (***bottom panel***) assuming a sample size of 151

The location parameter and the scaling parameter for the non-local prior are ${\mu}_{\delta}$ and $\tau$, respectively. The modes of the distribution are ${\mu }_{\delta} \pm \sqrt{2}\tau$. All distributions above are visualized in the top panel of Fig. 1, with the location and scaling parameters set to 0 and 0.707 (in line with the default in the BayesFactor R package), respectively.

### The design prior

When conducting BFDA, two kinds of priors need to be differentiated and specified (O’Hagan and Stevens, 2001; Schönbrodt & Wagenmakers, 2018; Walley et al., 2015). The first prior type is the aforementioned *analysis prior*. The analysis prior is the prior distribution used for calculating the BF once the data is collected, using Eqs. (3) or (4). The second kind of prior is the *design prior*, which is mainly used for the calculation of the probabilities of compelling and misleading evidence in Table 1, *before* data collection. Design priors are required for those wishing to conduct BFDA (Schönbrodt & Wagenmakers, 2018). The design prior should be informative, depending on the researcher’s prior knowledge and experience for designing an efficient study. In contrast, a default or “objective” analysis prior is often chosen for convincing a (wider range of) skeptical audience (Schönbrodt & Wagenmakers, 2018), particularly those who might be wary of the specified ‘subjective’ prior, or for merely following the convention of using a default prior (Pawel & Held, 2025). Nonetheless, the distinction between the two types of priors – analysis and design – is argued to be unnecessary and artificial when an informed prior is used, as both types reflect the prior belief on $\delta$ under the alternative hypothesis (Stefan et al., 2019, p. 1047).

Whether or not the two priors are specified identically, they serve different purposes in BFDA and in our sample size determination method. For clarity, the prior density function used for the analysis prior is denoted as ${\pi }_{a}\left(\delta \right)$, while the prior density function for the design prior is denoted as ${\pi}_{d}\left(\delta \right)$. To summarize, the analysis prior influences the calculation of the BF in Eqs. (3) and (4). The design prior, on the other hand, affects the computation of probabilities of compelling and misleading evidence within BFDA through the so-called prior predictive distributions, as explained next.

Applied researchers primarily interested in applying our method for BFDA may skip the following sections detailing our numerical method and proceed directly to section “The procedure and empirical application of BFDA”.

### Prior predictive distributions based on the design prior

Before data collection, the probability density for the unknown but observable *t*-values can be obtained using Eq. 1.3 in Gelman et al. (2013, p. 7):

7 $$p(t)=\int {T}_{v}\left(t|\sqrt{N}\delta \right){\pi}_{d}\left(\delta \right)d\delta .$$

This distribution of the observable *t*-values is the *prior predictive distribution* of the data conditional on the design prior density function ${\pi}_{d}\left(\delta \right)$. Unlike the prior distribution, which focuses on the effect size $\delta$, the prior predictive distribution emphasizes the distribution of the possible *t*-values before data collection. In the case of a one-sample *t* test with a point null hypothesis, the prior predictive distribution under the null hypothesis is simply the *t*-distribution with $p\left(t|{H}_{0}\right)={T}_{v}\left(t|0\right)$, as the null hypothesis simply assumes $\delta =0$ (in this case, the prior distribution is trivial and simply places all probability mass on the 0 value). As for the prior predictive distribution under the alternative hypothesis, the marginalized distribution of *t*-values can be found using Eq. (7) based on the specified design prior distribution on $\delta$. Equations (2) and (7) are similar, except that the analysis prior is used in the former equation. In the case of a point design prior, the prior predictive distribution can be simply obtained as ${T}_{v}\left(t|\sqrt{N}\delta \right).$ The lower panel in Fig. 1 shows how the *t*-values are distributed if $\delta$ follows different prior distributions, with a sample size of 151 and degrees of freedom equal to 150. The prior predictive distributions are generally more spread out than the prior distributions because the latter focus solely on the uncertainty of δ, while the former encapsulates uncertainty of both δ and the data. Next, we elaborate on how prior predictive distribution can be used for BFDA.

### Conducting BFDA using the prior predictive distributions

The first step of conducting BFDA is the specification of the design prior. Once a design prior is selected, the prior predictive distributions of the *t*-value under the null and alternative hypotheses can be determined. Following the previous example, in the case of a one-sample *t* test with a fixed sample of 151, if a Cauchy distribution with a scaling parameter of 0.707 is specified as the design prior, the prior predictive distribution under the alternative is shown in the lower panel of Fig. 1 with the solid line. Given a point null hypothesis, the prior predictive distribution of the *t*-values under the null hypothesis is the *t*-distribution.

**Fig. 2:**
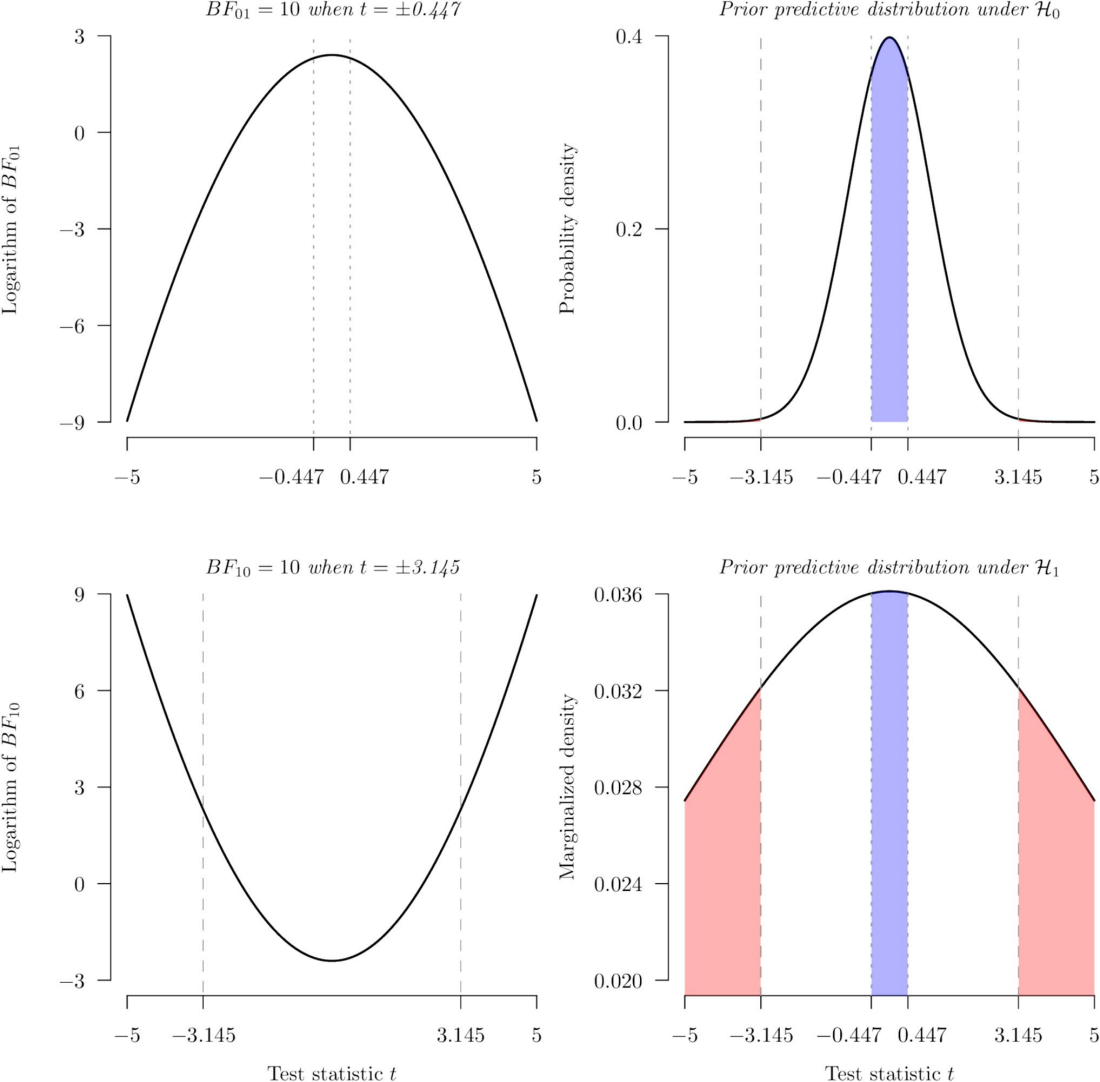
The procedure of finding the probability of compelling evidence and misleading evidence for a one-sample *t* test with* df* = 150. The *first column* demonstrates the relationship between the *t*-values and the natural logarithm of BF in favor of the null or the alternative based on (the inverse of) Eq. (3). The *second column* presents the predictive distributions, highlighting areas where the ${\text{BF}}_{01}$ (*blue*) or ${\text{BF}}_{10}$ (*red*) exceed 10

Our second step of BFDA is the specification of the analysis prior being used for the calculation of the BFs. The analysis prior influences the relationship between the *t*-values and the BFs, and the ranges of *t*-values associated with compelling BFs. Suppose the analysis prior is the same as the design prior. The relationship between the *t*-values and the natural logarithm of ${BF}_{10}$ (the second row) and ${BF}_{01}$ (the first row) is shown in the first column of Fig. 2 based on Eq. (3). The *uniroot* function from the stats R package and/or the *uniroot.all* from the rootSolve R package are used to find the *t*-values ${t}_{b}$ corresponding to a ${BF}_{b}$ of 10. The root-finding algorithm shows that when the *t*-value ${t}_{b01}$ is ± 0.447, the targeted strength of evidence ${BF}_{b}$ is achieved for ${BF}_{01}$. Similarly, when ${t}_{b10}$ is ± 3.145, the targeted strength of evidence ${BF}_{b}$ is achieved for ${BF}_{10}$.

Third, the probability of compelling evidence can be calculated using the following equations with the *integrate* function in R:

8 $$p\left({BF}_{10}>10|{H}_{1}\right)=\int \left({\int }_{-\infty }^{{t}_{b10}^{-}}{T}_{v}\left(t|\sqrt{n}\delta \right)dt+{\int}_{{t}_{b10}^{+}}^{\infty }{T}_{v}\left(t|\sqrt{n}\delta \right)dt\right){\pi }_{d}\left(\delta \right)d\delta =78.16\%,$$

9 $$p\left({BF}_{01}>10|{H}_{0}\right)={\int }_{{t}_{b01}^{-}}^{{t}_{b01}^{+}}{T}_{v}\left(t|0\right)dt=34.43\%.$$

The superscript plus sign (+) indicates the greater *t*-value of the two ${t}_{b}$, and the minus sign (-) indicates the smaller one. As for the probabilities of misleading evidence:

10 $$p\left({BF}_{10}>10|{H}_{0}\right)={\int }_{-\infty }^{{t}_{b10}^{-}}{T}_{v}\left(t|0\right)dt+{\int }_{{t}_{b10}^{+}}^{\infty }{T}_{v}\left(t|0\right)dt= 3.22\% ,$$

11 $$p\left({BF}_{01}>10|{H}_{1}\right)=\int {\int }_{{t}_{b01}^{-}}^{{t}_{b01}^{+}}{T}_{v}\left(t|\sqrt{n}\delta \right)dt{\pi }_{d}\left(\delta \right)d\delta =0.2\%.$$

The second column of Fig. 2 shows the prior predictive distributions under the null and the alternative hypotheses, highlighting the area where ${BF}_{10}$ (red) or ${BF}_{01}$(blue) are larger than 10. Thus, the red area represents the range of *t*-values leading to compelling evidence in favor of the alternative hypothesis relative to the null hypothesis, while the blue area indicates the *t*-values associated with compelling evidence for the null hypothesis relative to the alternative hypothesis. For instance, given that the alternative is true, the probability of obtaining compelling evidence for the alternative is around 78% with the bounds of *t*-values being ± 3.145. The above concludes the technical procedure of conducting BFDA for a fixed sample size of 151.

As for a priori BFDA for sample size determination, given the targeted probability of true-positive evidence $p\left({BF}_{10}>10|{H}_{1}\right)=80\%$ following the conventional β level of .2 in NHST, the *uniroot* function is used again to find the sample size *N* that satisfies this condition. A sample size of 186 is needed with:

$$\begin{array}{l}p\left({BF}_{10}>10|{H}_{1}\right)=80\%, p\left({BF}_{01}>10|{H}_{0}\right)=47.4\%,\\ p\left({BF}_{10}>10|{H}_{0}\right)= .2\%\, and\, p\left({BF}_{01}>10|{H}_{1}\right)=4.1\%.\end{array}$$

The approach above can easily be adapted for one-sided *t* tests and independent samples *t* tests with (un)balanced design (see the equations in Appendix 1 for details). The procedures and the equations described above have been integrated into a user-friendly Shiny app, which is showcased in the next section. When the design prior and analysis prior differ, small adjustments are necessary. The root-finding algorithm is based on the analysis prior in the second step because it influences the calculation of the BFs in Eq. (3) and the ranges of *t*-values associated with compelling BFs. On the other hand, the prior density function in Eqs. (8) through (10) should be based on the design prior in the third step, as the design prior determines the prior predictive distribution under the alternative hypothesis.

As Pawel and Held’s (2025) method only applies to situations in which the design prior is either a point or normal distribution and the analysis prior is either a scaled *t*-distribution or a non-local distribution, we can only compare our method with theirs while using similar specifications. Suppose the design prior and the analysis prior on $\delta$ are ${\pi}_{d}\left(\delta \right)= N\left({\mu }_{\delta }=0, {\sigma}_{\delta}^{2}={1}^{2}\right)$ and ${\pi}_{a}\left(\delta \right)=$ t(${\mu}_{\delta}=0$, $\lambda =.707$, $k=3$), respectively, for a two-sided independent samples *t* test. Table 2 shows the required sample sizes per group using the presented method and using the *ntbf01* function from Pawel and Held (2025) with various values for the probability of true-positive evidence $p\left({BF}_{10}>10|{H}_{1}\right)$. The results are identical. Note that the essential difference between the two methods is that Pawel and Held (2025, p.17) assumed normality of effect size estimates and known data variance (and thus known standard error of effect size estimates), leading approximately to $t \sim N\left({\mu}_{\delta }\sqrt{\frac{n}{2}} ,1+\frac{n{\sigma}_{\delta}^{2}}{2}\right)$ with the analytic solutions for obtaining the probability of true-positive evidence, using the standard normal cumulative function. Meanwhile, our method relies on numerical integration based on the marginalized cumulative probability of the *t*-distribution across all possible effect sizes under the normal (or any proper) design prior to obtain the cumulative probability of the predictive distribution. For this reason, when the sample size is sufficiently large, the results are expected to be consistent regardless of the normality assumption (as the *t*-distribution becomes the same as the standard normal distribution when the degrees of freedom approach infinity). However, a difference can be observed when the sample sizes are small. Table 3 illustrates this scenario by demonstrating that, as the sample size increases, the calculated probabilities of true-positive evidence obtained from the two methods converge to similar values. Our method has the advantage of offering greater flexibility in the specification of priors with (un)balanced group size while making less assumptions.

**Table 2:** The required sample size per group for a two-sided independent samples *t* test with a design prior N(μ_δ_ = 0, ${\sigma}_{\delta}^{2}$ = 1) and an analysis prior t (μ_δ_ = 0 , λ = .707, k = 3), using our method and Pawel and Held’s (2025) method

$p\left({BF}_{10}>10|{H}_{1}\right)$	0.5	0.55	0.6	0.65	0.7	0.75	0.8	0.85	0.9	0.95
The presented method	35	45	59	81	115	174	288	548	1348	6167
Pawel and Held (2025)	35	45	59	81	115	174	287	548	1347	6166

**Table 3:** The probability of compelling evidence for the alternative hypothesis, expressed as a percentage, with a design prior N (μ_δ_ = 0, ${\upsigma }_{\updelta }^{2}$ = 1) and an analysis prior t (μ_δ_ = 0 , λ = .707, k = 3), given various sample sizes per group, using our method and the analytical solutions by Pawel and Held (2025)

*N* per group	4	6	8	10	12	14	16	18	20	1000
The presented method	0.21	5.77	12.87	18.93	23.92	28.09	31.63	34.66	37.31	88.56
Pawel and Held (2025)	2.18	8.69	15.10	20.58	25.18	29.08	32.43	35.33	37.87	88.56

### The procedure and empirical application of BFDA

**Fig. 3:**
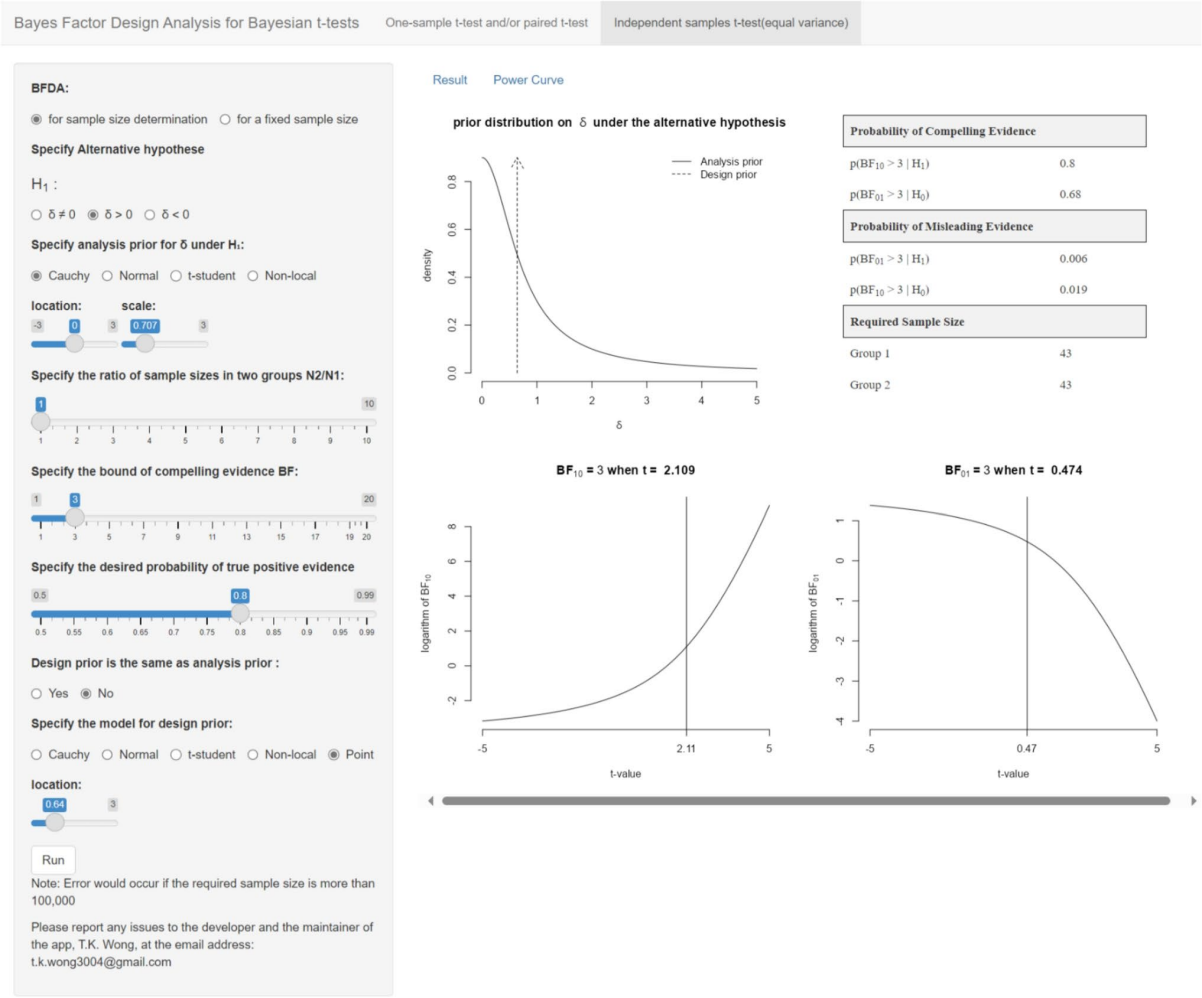
Screenshot of the Shiny app “Bayes factor design analysis for Bayesian *t* tests”

We developed a user-friendly Shiny app ( https://tkwong3004.shinyapps.io/BayesTtestPower/) that facilitates BFDA using the method outlined in the preceding section (Fig. 3). The source code is available at https://github.com/tkWong3004/BayesTtestPower. The process for determining sample size involves the following steps:

1. Specify the direction of the analysis and design priors. This direction is assumed to be the same, as it is illogical to have different or conflicting directionality between the two priors.
2. Specify the analysis and design prior distributions on δ. When the analysis and design priors differ, additional parameters need to be specified.

Optional: Specify the ratio of the sample size in group 2 over that in group 1 in the case of an independent-samples *t* test.

(3) Specify the targeted strength of evidence and the targeted probability of true-positive evidence.

Optional: Specify sample sizes (per group) instead of the probability of true-positive evidence when conducting BFDA for a fixed sample size.

Based on the input above, the tab “Result” in the Shiny app returns the needed sample size ensuring the informativeness and the efficiency of the study, or the app returns the evaluation of the study design with the probabilities of compelling and misleading evidence for a fixed sample size. Meanwhile, the tab “Power Curve” shows the relationship between the probability of the true-positive evidence and the sample size.

The available ranges for the specification of prior hyperparameters (e.g., location and scaling parameters) are set to accommodate common scenarios. If users wish to specify hyperparameter values beyond those allowed in the app, an R script “ScriptForBFDA.r” for conducting BFDA is available in the aforementioned GitHub repository. The readers are encouraged to explore our Shiny app and/or the R script with the following empirical application.

### Empirical application

Allen et al. (2021) developed a novel tool for training students in structural awareness. Structural awareness is defined as “the ability to look past the surface (or topic) features of a research design and focus instead on its deep structural characteristics” (Allen et al., 2021, p. 53). Students with better structural awareness understand the design of a study and can select appropriate statistics for various research designs. The researchers hypothesized that trained students would perform better than untrained students on different measures. Specifically, an effect size δ of 0.64 was specified with a fixed sample size of 102 and the targeted strength of evidence being ${BF}_{b}=3$ for BFDA (Allen et al., 2021, p. 55; Schönbrodt & Wagenmakers, 2018). The result of the BFDA for a one-sided Bayesian independent samples *t* test using simulation is (Allen et al., 2021, p. 55):

$$p\left(BF_{10}\;>\;\left.3\right|\;H_1\right)=86\%,\;p\left(\frac13<\;BF_{10}\;<\;\left.3\right|\;H_1\right)=14\%,\;p\left(BF_{01}\;>\;\left.3\right|\;H_1\right)<1\%.$$

Suppose the researchers wish to conduct a priori BFDA for sample size determination instead of having a fixed total sample size of 102. Our method would be useful in this scenario. First, a point design prior of 0.64 and a default analysis prior (i.e., Cauchy with a location parameter of 0 and a scaling parameter of .707) are specified following Allen et al. (2021) for an independent-samples *t* test with equal sample size per group. Second, the targeted strength of evidence and the targeted probability of true-positive evidence are set to 3 and 0.8, respectively. Using our Shiny app (see the top right panel of Fig. 3), the required sample size per group is determined to be 43. The *t*-values corresponding to a BF of 3 are shown in the lower panels of Fig. 3. Alternatively, BFDA for a fixed sample size of 102 can also be done with our app, leading to:

$$\begin{array}{lc}p\left(BF_{10}\;>\;\left.3\right|\;H_1\right)=86.3\%,\;p\left(BF_{01}\;>\;\left.3\right|\;H_1\right)=.04\%,\\p\left(\frac13\;<\left.BF_{10}\;<3\right|\;H_1\right)=1-86.3\%-.04\%=13.3\%.\end{array}$$

The result is similar to the ones obtained by Allen et al. (2021), and the difference between the results may be explained by rounding and simulation error.

Suppose we expect the true effect size to follow a one-sided *t*-distribution with a location parameter of 0 and a scaling parameter of 0.707, instead of a point design prior – matching the default analysis prior. In this case, a sample size of 200 per group is required. However, if the location parameter of the priors is set to 0.64, only 55 participants per group are needed.

In the context of meta-analysis, effect size is often assumed to follow a normal distribution to account for heterogeneity, representing the variability in effect sizes across different studies. Suppose a one-sided normal design prior is specified with a mean of 0 and a standard deviation of 1 and the default analysis prior, a sample size of 164 per group is required.

Lastly, suppose a one-sided non-local design and analysis prior with a location parameter of 0 and a scaling parameter of 0.45 (because the mode of the distribution is $\frac{.64}{\sqrt{2}}$) are specified to reflect the researchers’ expectations on the effect size. The required sample size per group is determined to be 48.

### Investigating the operational characteristics of the Bayesian *t* test

As demonstrated above, the required sample size varies depending on the analysis prior and design prior. This section aims to investigate and show how different prior specifications influence the operational characteristics of Bayesian *t* tests from a frequentist perspective, with a focus on controlling false-positive evidence (false-positive rate) and true-positive evidence (power) within the framework of statistical decision theory. Thus, the BF is used for reaching a decision depending on the bound ${BF}_{b}=b$: the alternative is “rejected” when ${BF}_{10}$ < $\frac{1}{b}$, the null is “rejected” when ${BF}_{10}$ > $b$ , otherwise no decision is made when $\frac{1}{b}<{BF}_{10}<b$.

**Fig. 4:**
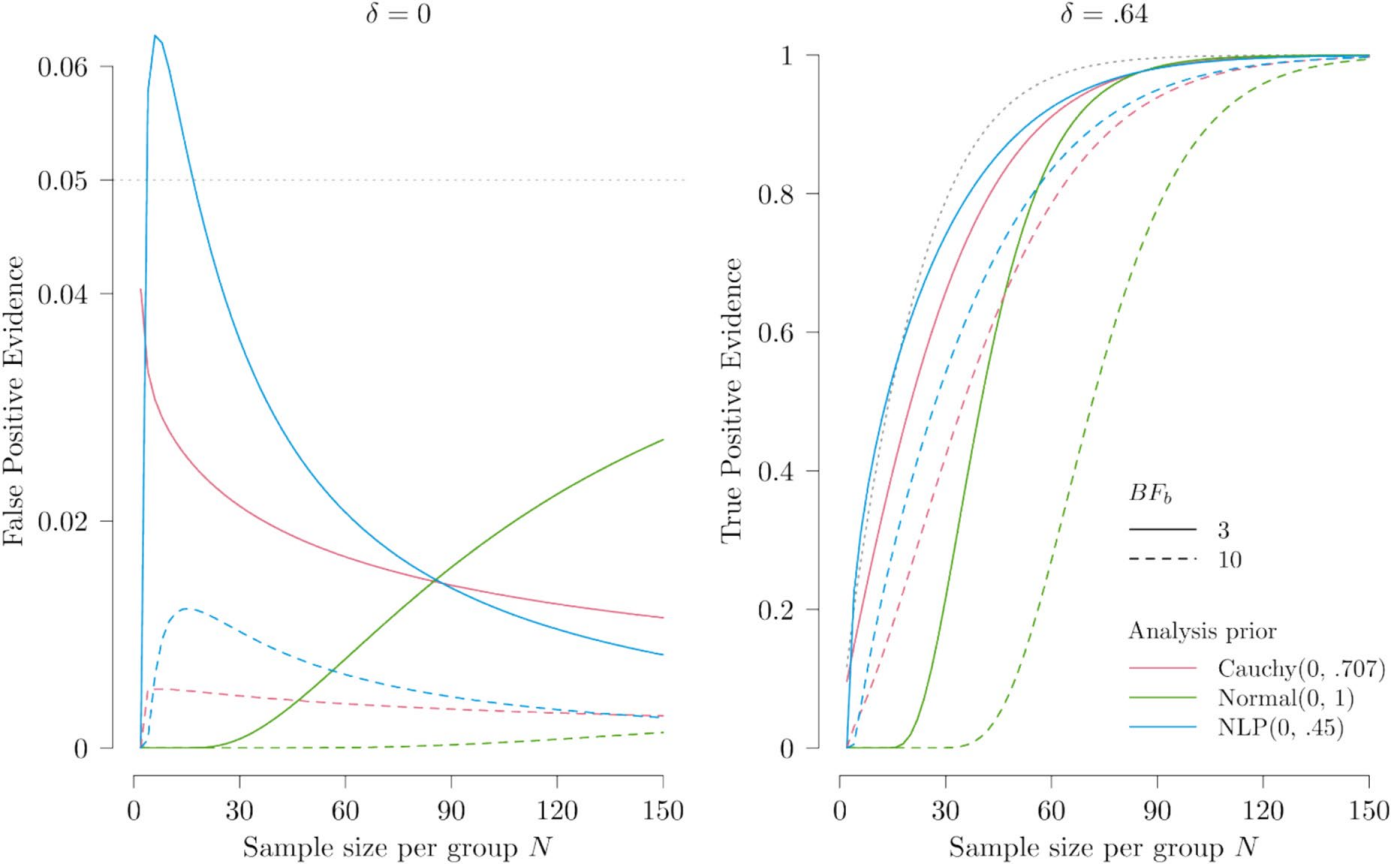
The operating characteristics of BFs with different analysis priors, sample sizes, and different bounds of compelling evidence given δ ∈ {0,0.64}

For our investigation, we examine the probability of false-positive evidence and true-positive evidence using the BF for decision-making with the Cauchy (.707), normal (0,1) , and non-local (0, .45) being the analysis priors, alongside a point design prior ∈ {0 , .64} in the context of a one-sided independent samples *t* test following the example from the previous section. Figure 4 illustrates the probability of obtaining ${BF}_{10}>{BF}_{b}$ with different analysis priors, given $\delta$ = 0 (left panel) or $\delta$ = 0.64 (right panel). The pink line represents the Cauchy prior, the green line represents the normal prior, and the blue line represents the non-local prior. The bounds of compelling evidence are indicated by the dotted line for ${BF}_{b}=3$ and the solid line for ${BF}_{b}=10$. The left panel shows the probability of false-positive evidence (i.e., α from a frequentist perspective), while the right panel shows the probability of true-positive evidence (i.e., power).

Generally, the probabilities of false-positive evidence are well below the conventional α level of 0.05, decreasing as sample size increases for the Cauchy and non-local priors. The exceptions are the non-local analysis prior with small sample sizes and the normal analysis prior. Meanwhile, the probability of true-positive evidence with the non-local analysis prior increases at a faster rate as sample size increases compared to the Cauchy and normal priors. Thus, the non-local prior is more efficient than other priors as it allows more rapid accumulation of evidence for the alternative, similar to the findings in Pramanik and Johnson (2024). Furthermore, the power curves for all analysis priors are lower than the power curve (gray dotted line) obtained from NHST with α = 0.05. Finally, higher bounds for compelling evidence are associated with lower probabilities of false-positive evidence and true-positive evidence as indicated by the difference between the dotted line and solid line.

**Fig. 5:**
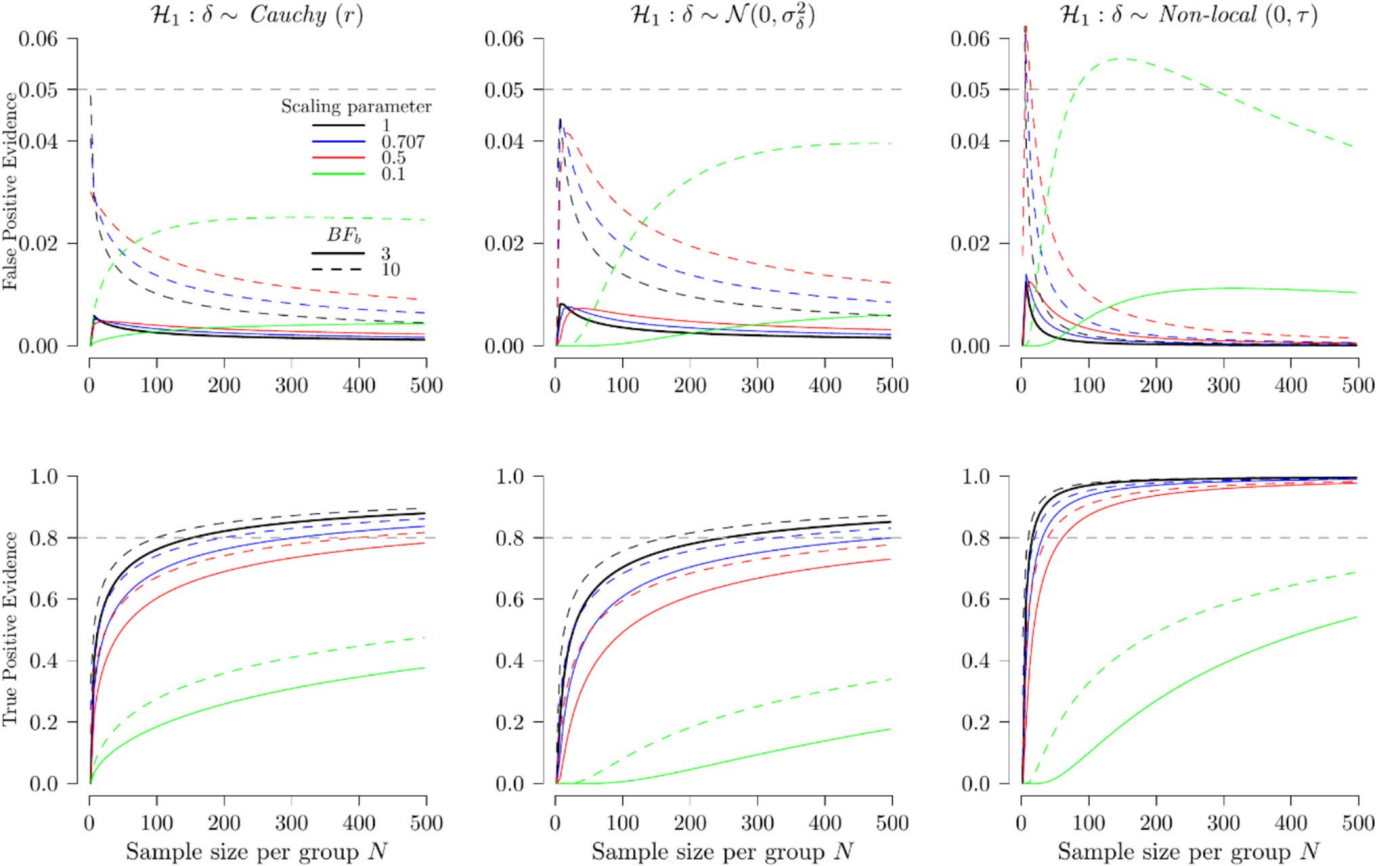
The operating characteristics of BFs with different prior distributions, scaling parameters, sample sizes, and different bounds of compelling evidence. Note*: r*, σ and τ ∈ {.1, .5, .707, 1} with color ∈{*green*, *red*, *blue*, *black*}. ${BF}_{b}$ ∈ {3, 10} with type of line ∈{*dotted line*, *solid line*}. A colorblind-friendly version of the plot is available at https://github.com/tkWong3004/BayesTtestPower

Further investigation is conducted with a broader range of prior specifications rather than focusing solely on the point design prior. We examine the probability of false-positive evidence with the Cauchy (*r*), normal (0, ${\sigma }_{\delta }^{2}$) , and non-local (0, τ) being the analysis priors, where *r*, ${\sigma }_{\delta }$ and τ ∈ {1, 0.707, 0.5, 0.1} alongside a point-null design prior, in the context of a one-sided independent samples *t* test. The first row of Fig. 5 illustrates the probability of false-positive evidence for different analysis priors and scaling parameter values: black for 1, blue for 0.707, red for 0.5, and green for 0.1. The bounds for compelling evidence are indicated by the dotted line for a BF of 3 and the solid line for a BF of 10. Meanwhile, the second row of Fig. 5 shows the probability of true-positive evidence when the design and the analysis prior are the same.

As for the effect of sample size, the probabilities of false-positive evidence similarly decrease as the sample size increases and remain well below the conventional α level of 0.05. One exception is the non-local prior, where the probability exceeds 0.05 when the sample size per group is too small, given that ${BF}_{b}=3$. When the scaling parameter is small (green line) and close to the null hypothesis in terms of statistical models, the probabilities follow a different trend: they increase with a larger sample size until a certain threshold, after which they begin to decrease. Meanwhile, the probability of true-positive rate increases with increasing sample size.

As for the influence of the priors, we observe that the non-local prior results in a higher probability of false-positive evidence and a greater (or more rapidly increasing) probability of true-positive evidence compared to the Cauchy and normal priors. These observations illustrate that the trade-off between the probability of misleading and compelling evidence parallels the trade-off between α and power in NHST.

However, this trade-off does not necessarily hold when examining the effect of the scaling parameter within a given prior. Specifically, a higher scaling parameter (black and blue) is associated with a lower probability of false-positive evidence and a higher probability of true-positive evidence compared to a lower scaling parameter (red and green). This may be because a BF with a wider analysis prior results in a higher ${t}_{b}$ (e.g., ${t}_{b}$ = 2.334 when scaling parameter = .707, but ${t}_{b}$ = 2.441 with scaling parameter of 3 given ${BF}_{b}=3$). Consequently, the probability of false-positive evidence decreases as the scaling parameter increases. The higher probability of true-positive evidence may be attributed to the fact that a design prior with a larger scaling parameter assigns more density to nonzero effect sizes, making it more likely to obtain a compelling BF.

When compared to NHST, the Bayesian independent samples *t* test is generally more severe than NHST with conventional α = .05, similar to the findings of Kelter (2021) using simulation and Pawel and Held (2025). Moreover, compelling BFs (i.e., ${BF}_{10}$ > 3 or 10) are almost always associated with significant *p* values. However, significant *p* values do not necessarily correspond to compelling BFs. Nonetheless, the increased severity comes at the cost of a decrease in power. Furthermore, when using BFs for decision-making as a frequentist procedure, the false-positive rates and power are automatically adjusted with increasing sample size​. In contrast, NHST maintains a fixed α level with increasing power. Additionally, power analysis in NHST typically assumes a fixed effect size, even though effect size heterogeneity is common in meta-analysis (Linden & Hönekopp, 2021; Van Erp et al., 2017). This heterogeneity suggests that effect sizes vary across studies. By incorporating a prior distribution, the Bayesian approach accounts for this variability (Pek & Park, 2019).

## Discussion

Methods using root-finding algorithms for sample-size determination overcome the difficulty of conducting BFDA and facilitate the use of BFDA among applied researchers without the need of simulation (Pawel & Held, 2025; Weiss, 1997). In this paper, we developed an alternative method that generalizes the approach by Pawel and Held (2025). Our method requires less statistical assumptions (e.g., the normality assumption for the effect size estimate) and allows for more flexibility in the specification of the design and analysis priors. Besides the a priori BFDA for sample size determination, BFDA for a given sample size for (un)balanced design is available as well without simulation. A user-friendly Shiny app is also made available for conducting BFDAs without requiring any programming skills from the user. Moreover, we provide additional insights into the operating characteristics of Bayesian *t* tests and NHST within the framework of statistical decision theory, verifying results in existing literature (e.g., Kelter, 2021; Pawel & Held, 2025; Pramanik & Johnson, 2024).

As for the limitation of our method and the Shiny app, our approach is not applicable to BFs where the analysis prior depends on observed data, such as fractional BFs (O’Hagan, 1995) or Welch’s *t* test (as its degree of freedom depends on observed variances), as such information cannot be known before data collection. Future research should address sample size determination for these types of BFs and for Welch’s *t* test. Regarding the Shiny app, the app can only calculate the required sample sizes up to 100,000 (per group) for stable results. Nevertheless, this is a reasonable constraint, as it is practically unlikely for a research design to require a sample size of this magnitude. More specifically, this limitation is most relevant when the design and/or analysis prior assigns most of the density on $\delta$ near the null value, or when the analysis prior is uninformative, characterized by large scaling parameters (i.e., a larger value of *r*, ${\sigma }_{\delta }$ or τ for analysis prior with a smaller value of these parameters for the design prior) with a high specified probability of true-positive evidence. The above situation is analogous to a frequentist power analysis with a specified effect size smaller than expected (e.g., specifying $\delta$ = .01 instead of .2) and great desired power (e.g., 99%). The direct consequence would be the increase in the “true” power (e.g., 99.99%). Thus, specifying a design prior with a small scaling parameter, suggesting that the effect sizes are much closer to zero than expected, leads to an increase in the ‘true’ probability of true-positive evidence. Nevertheless, such a specification might go against the purpose of BFDA, which aims to use the smallest possible sample size for informative results.

Moreover, our method is limited to a fixed-*n* research design, where the sample size is determined before data collection. It cannot be applied to a sequential design (Wald, 1945), in which data is collected continuously until a compelling BF is obtained, since the likelihood function of the effect size estimates is unknown. For this reason, readers are referred to the simulation methods proposed by Schönbrodt and Wagenmakers (2018) and Stefan et al. (2019).

Another limitation is the restricted range of BFDA applications, as our implementations are currently limited to *t* tests. However, we provide a solid foundation for future research on sample size determination, which can be extended to other statistical tests without the use of simulation and with less statistical assumptions. The current method can be extended to testing scenarios with two (truncated) composite hypotheses as well and other tests (e.g., proportions, ANOVA, and regression). Ideally, a user-friendly program similar to G*Power (Faul et al., 2007) encompassing a wide range of tests may be developed in the future. At the moment, readers who wish to conduct BFDA involving a z-test, correlations, or log odds are referred to Pawel and Held (2025) and the binomial setting to Kelter and Pawel (2025). We hope our paper and Shiny app further facilitate the use of BFDA among applied researchers design studies yielding informative results using minimal resources and facilitating future research on BFDA.

## Data Availability

Not applicable

## Appendix 1

The equations of the probability of compelling and misleading evidence for the one-sided, one-sample and paired *t* tests are as follows, with ${t}_{b10}$ being the *t*-value when ${BF}_{10}=b$ , $v=N-1$, $\pi \left(\delta \right)= \frac{f\left(\delta \right)}{F\left(A\right)-F(B)}$ for one-sided tests and, the upper and lower bounds of the integral being A and B, respectively:

12 $$p\left({BF}_{10}>b\vert H_1\right)=\left\{\begin{array}{lc}\int_0^\infty\int_{t_{b10}}^\infty T_v\left(t\vert\sqrt n\delta\right)dt\pi\left(\delta\right)d\delta,\;\;\;\;\;\;\;\;\;when\;H_1:\delta>0,\\\int_{-\infty}^0\int_{-\infty}^{t_{b10}}T_v\left(t\vert\sqrt n\delta\right)dt\pi\left(\delta\right)d\delta,\;\;\;\;\;\;\;when\;H_1:\delta<0,\end{array}\right.$$

13 $$p\left({BF}_{01}>b|{H}_{0}\right)= \left\{\begin{array}{lc}{\int }_{-\infty }^{{t}_{b01}}{T}_{v}\left(t|0\right)dt, \ \ \ \ \ \ \ \ \ when\ {H}_{1}: \delta >0,\\ {\int }_{{t}_{b01}}^{\infty }{T}_{v}\left(t|0\right)dt, \ \ \ \ \ \ \ \ \ when\ {H}_{1}: \delta <0,\end{array}\right.$$

14 $$p\left({BF}_{10}>b\vert H_0\right)=\left\{\begin{array}{lc}\int_{t_{b10}}^\infty T_v\left(t\vert0\right)dt,\;\;\;\;\;\;\;\;\;when\;H_1:\delta>0,\\\int_{-\infty}^{t_{b10}}T_v\left(t\vert0\right)dt,\;\;\;\;\;\;\;\;\;when\;H_1:\delta<0,\end{array}\right.$$

15 $$p\left({BF}_{01}>b\vert H_1\right)=\left\{\begin{array}{lc}\int_0^\infty\int_{-\infty}^{t_{b01}}T_v\left(t\vert\sqrt n\delta\right)dt\pi\left(\delta\right)d\delta,\;\;\;\;\;\;\;\;\;when\;H_1:\delta>0,\\\int_{-\infty}^0\int_{t_{b01}}^\infty T_v\left(t\vert\sqrt n\delta\right)dt\pi\left(\delta\right)d\delta,\;\;\;\;\;\;\;\;\;when\;H_1:\delta<0.\end{array}\right.$$

The ones for independent samples *t* test are the following with $v= {n}_{1}{ + n}_{2}-2$:

16 $$p\left({BF}_{10}>b|{H}_{1}\right)=\left\{\begin{array}{lc}{\int }_{0}^{\infty }{\int }_{{t}_{b10}}^{\infty }{T}_{v}\left(t|\sqrt{\frac{{n}_{1}{n}_{2}}{{n}_{1}{+n}_{2}}}\delta \right)dt\pi \left(\delta \right)d\delta , \ \ \ \ \ \ \ \ \ \ \ \ \ \ \ \ \ \ \ \ \ \ \ \ \ \ \ \ \ \ \ \ \ \ \ \ \ \ \ \ \ \ \ \ \ \ \ \ \ \ \ \ \ \ when\ {H}_{1}: \delta >0,\\ \int \left({\int }_{-\infty }^{{t}_{b}^{-}}{T}_{v}\left(t|\sqrt{\frac{{n}_{1}{n}_{2}}{{n}_{1}{+n}_{2}}}\delta \right)dt+{\int }_{{t}_{b}^{+}}^{\infty }{T}_{v}\left(t|\sqrt{\frac{{n}_{1}{n}_{2}}{{n}_{1}{+n}_{2}}}\delta \right)dt\right)\pi \left(\delta \right)d\delta , \ \ \ \ \ \ \ \ \ {when}\ { H}_{1}: \delta \ne 0\\ {\int }_{-\infty }^{0}{\int }_{-\infty }^{{t}_{10}}{T}_{v}\left(t|\sqrt{\frac{{n}_{1}{n}_{2}}{{n}_{1}{+n}_{2}}}\delta \right)dt\pi \left(\delta \right)d\delta , \ \ \ \ \ \ \ \ \ \ \ \ \ \ \ \ \ \ \ \ \ \ \ \ \ \ \ \ \ \ \ \ \ \ \ \ \ \ \ \ \ \ \ \ \ \ \ \ \ \ \ \ \ {when}\ { H}_{1}: \delta <0,\end{array}\right.$$

18 $$p\left({BF}_{01}>b|{H}_{0}\right)= \left\{\begin{array}{lc}{\int }_{-\infty }^{{t}_{b01}}{T}_{v}\left(t|0\right)dt, \ \ \ \ \ \ \ \ \ when \ {H}_{1}: \delta >0,\\ {\int }_{{t}_{b}^{-}}^{{t}_{b}^{+}}{T}_{v}\left(t|0\right)dt, \ \ \ \ \ \ \ \ \ \ {when}\ {H}_{1}: \delta \ne 0,\\ {\int }_{{t}_{b01}}^{\infty }{T}_{v}\left(t|0\right)dt, \ \ \ \ \ \ \ \ \ when\ {H}_{1}: \delta <0,\end{array}\right.$$

19 $$p\left({BF}_{10}>b|{H}_{0}\right)=\left\{\begin{array}{lc}{\int }_{{t}_{b10}}^{\infty }{T}_{v}\left(t|0\right)dt, \ \ \ \ \ \ \ \ \ \ \ \ \ \ \ \ \ \ \ \ \ \ \ \ \ \ \ \ \ \ \ \ \ \ \ \ when\ {H}_{1}: \delta >0,\\ {\int }_{-\infty }^{{t}_{b}^{-}}{T}_{v}\left(t|0\right)dt+{\int }_{{t}_{b}^{+}}^{\infty }{T}_{v}\left(t|0\right)dt, \ \ \ \ \ \ \ \ when\ {H}_{1}: \delta \ne 0\\ {\int }_{-\infty }^{{t}_{b10}}{T}_{v}\left(t|0\right)dt, \ \ \ \ \ \ \ \ \ \ \ \ \ \ \ \ \ \ \ \ \ \ \ \ \ \ \ \ \ \ \ \ \ \ \ \ when\ {H}_{1}: \delta <0,\end{array},\right.$$

20 $$p\left({BF}_{01}>b\vert H_1\right)=\left\{\begin{array}{lc}\int_0^\infty\int_{-\infty}^{t_{b01}}T_v\left(t\vert\sqrt{\frac{n_1n_2}{n_1{+n}_2}}\delta\right)dt\pi\left(\delta\right)d\delta,\;\;\;\;\;\;\;\;\;when\;H_1:\delta>0,\\\int\int_{t_b^-}^{t_b^+}T_v\left(t\vert\sqrt{\frac{n_1n_2}{n_1{+n}_2}}\delta\right)dt\pi\left(\delta\right)d\delta,\;\;\;\;\;\;\;\;\;\;\;\;\;when\;H_1:\delta\neq0,\\\int_{-\infty}^0\int_{t_{b01}}^\infty T_v\left(t\vert\sqrt{\frac{n_1n_2}{n_1{+n}_2}}\delta\right)dt\pi\left(\delta\right)d\delta,\;\;\;\;\;\;\;\;when\;H_1:\delta<0.\end{array}\right.$$

Note: The integrals under the null hypothesis (12, 13, 18, and 19) or a point prior can be obtained using the cumulative distribution function without numerical integration.

## Code availability

The R code for the figures, tables, and the Shiny app are available at https://github.com/tkWong3004/BayesTtestPower.
